# Knowledge, behaviour, and policy: questioning the epistemic presuppositions of applying behavioural science in public policymaking

**DOI:** 10.1007/s11229-021-03026-6

**Published:** 2021-02-05

**Authors:** Magdalena Małecka

**Affiliations:** 1 Faculty of Social Sciences, University of Helsinki, Unioninkatu 40A, P.O. box 24, 00014 Helsinki, Finland; 2 School of Social Science, Institute for Advanced Study, 1 Einstein Drive, Princeton, NJ 08540 USA

**Keywords:** Philosophy of behavioural science, Behavioural sciences in policy, Values in science, Behavioural economics, Cognitive psychology, Knowledge production in behavioural research, Feminist philosophy of science

## Abstract

The aim of this article is to question the epistemic presuppositions of applying behavioural science in public policymaking. Philosophers of science who have examined the recent applications of the behavioural sciences to policy have contributed to discussions on causation, evidence, and randomised controlled trials. These have focused on epistemological and methodological questions about the reliability of scientific evidence and the conditions under which we can predict that a policy informed by behavioural research will achieve the policymakers’ goals. This paper argues that the philosophical work of Helen Longino can also help us to have a better and fuller understanding of the knowledge which the behavioural sciences provide. The paper advances an analysis of the knowledge claims that are made in the context of policy applications of behavioural science and compares them with the behavioural research on which they are based. This allows us to show that behavioural policy and the debates accompanying it are based on an oversimplified understanding of what knowledge behavioural science actually provides. Recognising this problem is important as arguments that justify reliance on the behavioural sciences in policy typically presume this simplification.

## Introduction

Recently, in both policy circles and academia, there has been increasing interest in using insights from the behavioural sciences in order to inform policymaking. Policies inspired by findings in behavioural research are often called ‘behavioural public policies’. Their proponents claim that the behavioural sciences provide us with knowledge that could and should be used to design public policies. They believe that important societal problems and challenges, such as addiction, obesity, decreasing retirement savings, unsustainable consumption patterns, poverty, and even epidemics, such as the recent COVID-19, can be effectively addressed by interventions informed by behavioural research (Shafir 2012; Oliver 2013; Thaler and Sunstein 2008; Sunstein 2016; Chetty 2015; Bavel et al. 2020).

In the discussion on behavioural public policy, the most debated philosophical treatments can be found within moral and political philosophy.^1^ The work proposed by philosophers of science, including philosophers of the social sciences, has mainly contributed to discussions on causation, evidence, and randomised controlled trials (Grüne-Yanoff 2016; Grüne-Yanoff and Hertwig 2016; Marchionni and Reijula 2019), all of which have focused on epistemological and methodological questions about the reliability of scientific evidence and on asking about the conditions under which we can predict that a policy informed by behavioural research will achieve a goal which policymakers would set for themselves. Philosophers of science ask these questions inspired by philosophical work on evidence-based policy (e.g. Cartwright and Hardie 2012) and their research is meant to contribute to the analysis of how policies impact behaviour.^2^ In doing so, however, they do not scrutinise the kind of knowledge that is provided by ‘behavioural insights’ entering policy setting.

Therefore, there is a need for another type of analysis in philosophy of science in the context of behavioural policy, which I attempt to advance. The analytical strategy employed in this paper has been inspired by Helen Longino’s most recent book (Longino 2013). Longino examined several approaches in behavioural studies on aggression and sexuality and she argued that once we understand how empirical research on behaviour is produced, we will notice that it does not offer us knowledge which those who rely on this research in practical contexts believe they have. In a similar spirit, I analyse knowledge claims that are made in the context of policy applications of behavioural science (and which are a basis for policy proposals) and I compare them with the behavioural research on which they are based. This allows me to show that behavioural policy and the debates accompanying it are based on an oversimplified understanding of what knowledge behavioural science provides. Recognising this problem is important, because arguments which justify reliance on the behavioural sciences in policy presume and depend on this oversimplification, as do the points made by critics of behavioural policy.

As part of my analysis, I draw upon insights from feminist philosophy of science to ask what knowledge the behavioural sciences, widely used in policymaking, provide. Feminist philosophers of science are mainly recognised for their contribution to uncovering how gender preconceptions enter scientific research at each stage of inquiry (e.g. Keller and Longino 1996). Yet it should be noted that at the same time they also propose an approach to analysing models, experimental findings, and background assumptions that are indispensable when making sense of evidence (e.g. Longino 1990, 2013; Anderson 2004). It is this approach, and not focus on gender assumptions, which has informed my analysis in the project. In order to understand what we know on the basis of behavioural science it is not enough to look only at evidence. We should also reconstruct how relationships between phenomena are conceptualised in background assumptions (Longino 1990), and how behaviours are operationalised when studied experimentally (Longino 2013).

Feminist philosophers of science have inspired a lively discussion on values in science, which is also relevant to my analysis. In contemporary philosophy of science there is a widely accepted consensus that so-called non-epistemic values—political, social and ethical ones—cannot be separated from the processes of scientific knowledge production (Douglas 2009; Longino 1990; Wylie and Nelson 2007; Anderson 2004; Elliott 2017). They interfere with what evidence is available and whether or not it is regarded as reliable (Douglas 2009), or relevant (Longino 1990); values enter research via background assumptions adopted by researchers in order to make sense of data (Longino 1990) and the concepts they choose to employ (Dupré 2007); values also influence the ways in which scientific claims are justified (Intemann 2001). In addition, value commitments can impact choices about the acceptance of hypotheses and theories. For example, non-epistemic values are often relied upon when scientists assess the consequences of making a mistake while deciding about the evidential support for a hypothesis (Rudner 1953; Douglas 2000). It is widely debated which forms of value-ladenness are legitimate, or illegitimate (Douglas 2009; Elliott 2017), as well as what are the ethical, political, and policy implications of values in science (Douglas 2009; Biddle 2018; Tuana 2010). In order to analyse knowledge claims about the behavioural sciences I rely mainly on Helen Longino’s insights on the presence of values in science.

In this paper, I start by providing background information on the origins and development of behavioural public policy (Sect. 2). Then, I analyse the knowledge claims about the behavioural sciences widely accepted by proponents, discussants and many opponents of the behavioural policy. These scholars claim that behavioural research offers ‘descriptive’ and ‘realistic’ views on decision-making, that it reveals the irrationality of human behaviour, that it identifies cognitive causes of behavioural changes, and that it uncovers behavioural tendencies which are systematic and robust (Sect. 3). In order to examine each of these claims critically, I analyse knowledge provided by prospect theory, heuristics and biases programme, and research on loss aversion—a subset of behavioural research that is ubiquitously referred to in order to design and justify behavioural public policy (Sect. 4). Later (Sect. 5), I compare what we know on the basis of this research with the knowledge claims made by proponents of behavioural policy, endorsed widely also in the debates accompanying behavioural public policy. My aim is to show that these claims oversimplify and misinterpret knowledge provided by the behavioural sciences. In order to make this point I rely on philosophical insights from feminist philosophers of science (such as Longino) and on historical works on the origins of the behavioural sciences. In particular I argue that the oversimplification of what we know based on the behavioural sciences, which is prevalent in the debates on behavioural public policy, has come from ignoring and not accounting for the consequences of the underdetermination of theory by evidence, the value-ladenness of behavioural research, the history of the behavioural sciences, and scientific disagreement in behavioural research. Finally (Sect. 6), I formulate further questions which my analysis provokes.

## What is behavioural public policy?

Behavioural public policy gained momentum around twelve years ago after the publication of the book *Nudge—Improving Decisions about Health, Wealth, and Happiness* (Thaler and Sunstein 2008). The book not only advocated reliance on the behavioural sciences in policy, but it also promoted a new policy tool and approach: nudging. Essentially, nudging is a technique of a behaviour change used in policy contexts and is justified from the point of view of libertarian paternalism (Thaler and Sunstein 2008).^3^ A variety of policy fields such as health and environmental policy, consumer protection, and retirement savings are changing under the influence of the behavioural sciences, and the so-called behavioural turn in policy is reshaping public policy around the world (Jones et al. 2013).^4^

Reliance on behavioural research in policy is advocated as a way of making policies effective and as a remedy for the dominance and flaws of neoclassical economics as a policy-relevant scientific discipline (Shafir 2012; Hansen and Jespersen 2013). The view of human behaviour as driven by maximisation of expected utility has had an important impact on how policies were designed and conceptualised—as incentives to which policy addressees ‘rationally’ reply or react (e.g. Weimer and Vining 2017). Yet neoclassical economics has been challenged as an epistemic project, in particular by advocates of behavioural economics. Behavioural economics, inspired by cognitive psychology, is viewed as an alternative to neoclassical economics (or as a modification of it) (see e.g. Thaler 2000; Camerer and Loewenstein 2004; Angner 2012; Wilkinson and Klaes 2012). Behavioural economics and cognitive psychology are perceived as a source of scientific knowledge about behaviour that could provide a better, more accurate basis for designing policies (Oliver 2013, Thaler and Sunstein 2008). Furthermore, there is a more general argument in light of which behavioural public policy is advocated and accepted. It is widely believed that to design policies, policymakers should know how the people to whom policies are addressed behave, and why they behave in the way they do. Otherwise, they risk introducing ineffective policies, that is, policies which don’t bring about an outcome envisioned by them. Hence, proponents and enthusiasts of relying on scientific evidence in policymaking believe that in order to perform their societal function better, policymakers should be informed in particular by the sciences of behaviour (see e.g.: Shafir 2012; Marchionni and Reijula 2019).

For instance, evidence concerning status quo bias^5^ is brought up to advocate for default (opt-out) rules. Adherents of behavioural policy claim that because people exhibit status quo bias, some socially and policy relevant choices (e.g. organ donation, retirement savings) could be introduced as opt-out rules. For example, if people are automatically enrolled in retirement saving programmes, many more people tend to stay in the programme, due to the status quo bias, than if they were asked to actively enrol in it (Thaler and Sunstein 2008).^6^ Another example of behavioural science findings applied to policy is research on availability heuristics and affect heuristics.^7^ It is argued that a policymaker should take into account the fact that people typically rely on information available, visible, often emotionally laden, and easily retrievable from memory when they make decisions and assess the probability of events. The belief is that policymakers should use knowledge about this behavioural tendency when designing public health policy that discourages unhealthy activities (such as smoking) by means of placing vivid and shocking warnings on cigarette packets. Proponents of such a policy solution claim that due to the reliance on the availability heuristic and affect heuristic, people will overestimate the probability of the negative consequences of smoking, depicted on warnings, and engage in unhealthy activity less frequently (Peters 2011; Finucane et al. 2000; Slovic et al. 2005).^8^

## Knowledge claims about research in the behavioural sciences

‘The behavioural sciences’ in the context of nudging and behavioural policy capture a narrow subset of behavioural research. Behavioural public policy draws on selected findings in cognitive psychology and behavioural economics, such as the prospect theory, the heuristics-and-biases programme, social preferences, and empirical research inspired by these theoretical proposals. Proponents of behavioural public policy and those who debate the potential and limitations of behavioural policy seem to agree that cognitive psychology and behavioural economics reveal ‘irrationalities’ of behaviour and give us a more ‘realistic’, or more ‘descriptive’ view of human behaviour, compared to neoclassical economics (in particular to the expected utility theory, henceforth EUT^9^). This idea is expressed in the debates about behavioural policy in the following way:

research by psychologists and economists over the past three decades has raised questions about the rationality of many judgments and decisions that individuals make. People fail to make forecasts that are consistent with Bayes's rule, use heuristics that can lead them to make systematic blunders, exhibit preference reversals (…), suffer from problems of self-control, and make different choices depending on the framing of the problem (Sunstein and Thaler 2003: p. 1168);

…behavioural economics challenges all of these assumptions [of neoclassical economics—MM] and attempts to replace them with more realistic approaches based on scientific findings from other social sciences (Jolls et al. 1998: p. 1525);

Behavioural policy (…) is premised on the idea that interventions in public policy should be based on a psychologically realistic picture of human behaviour and its causes (Marchionni and Reijula 2019: p. 56).

Adherents of behavioural policy, as well as some behavioural scientists also claim that this research identifies in experimental settings robust phenomena—systematic tendencies of behaviour, such as loss aversion, framing effect, reliance on heuristics. The behavioural tendencies, some of which are called biases, concern the ways in which people make judgments about the probability of events, how they make decisions, and how they draw statistical and logical inferences. For instance, Sunstein and Thaler (2008) claim that ”[h]undreds of studies confirm that human forecasts are flawed and biased” (7) and Kahneman et al. (1991) argue that “[a]fter more than a decade of research on this topic we have become convinced that the endowment effect, status quo bias, and the aversion to losses are both robust and important” (205).

Furthermore, proponents of behavioural policy claim that biases are caused by cognitive processes guiding information processing, sometimes called “psychological principles that underlie human behaviour” (Sunstein and Thaler 2008: p. 112). This belief also takes the following shape in the literature and in the debates that behavioural policy has provoked:

experimental evidence pioneered in cognitive and social psychology show[s] that much of our individual and social behaviour is due to our brains processing information in ways that are not only bounded but also cognitively biased, where a cognitive bias is a systematic pattern of deviation in judgment or decision-making (Hansen 2016: p. 7);

Nudges co-opt the decision maker’s (internal) cognitive and motivational processes and design the (external) choice architecture such that it, in tandem with the (untouched) functional processes, produces a change in behaviour (Grüne-Yanoff and Hertwig 2016: p. 979).

Biases and irrationality are treated as problematic consequences of people’s behaviour that should be accounted for in policy settings. For instance, Guala and Mittone 2015 point out that “[n]udge policies typically remove psychological biases that prevent people from making the right decisions, or use the biases to direct behaviour towards better options” (385)*.*^10^

To summarise, then, we see that a set of knowledge claims about behavioural research, widely shared among proponents of behavioural public policy and in the debates accompanying it, has several characteristics. In particular, it is claimed that behavioural science identifies (1) causes of behaviour by cognitive processes, and (2) systematic behavioural tendencies that are a manifestation of irrationality; it is also believed that (3) behavioural theories of decision-making (in particular prospect theory) are more descriptive and realistic than the expected utility theory and that (4) behavioural experimental and empirical research identifies robust phenomena.

My aim is to examine whether this view of what we know on the basis of the behavioural sciences holds after we analyse knowledge provided by research in the behavioural sciences. Therefore, in the following sections of the article I compare the above-mentioned knowledge claims about the behavioural sciences with the behavioural research itself. I then argue that the claims about behavioural science which the proponents of behavioural policy make are not really substantiated in behavioural research. If that is the case, the project of behavioural public policy is significantly weakened and challenged, as it is justified by presuming knowledge claims that are questionable.

## What do we know?

In what follows, I draw upon Longino’s philosophical work on behavioural research. In her most recent book (Longino 2013), she pioneered a philosophical strategy and analysis that I employ here. She noticed that in the so-called nature/nurture debate, it is presumed that empirical studies in the behavioural sciences inform us about contributing causal factors of behaviour (aggression or homosexual practices) and that it is believed that they can be identified either at the ‘level‘ of genes, hormones, or the social environment. Longino looked at the empirical research in the behavioural sciences on aggression and sexuality (produced within quantitative behavioural genetics, social-environmental approaches, molecular behavioural genetics, neurobiological approaches, and integrative approaches) and she asked: what knowledge do these various approaches actually provide? She endeavoured to understand what we can learn about the causal factors of aggression or sexuality on the basis of knowledge accumulated within this research. She argues that each of the five approaches represents the causal space differently and we cannot integrate them to achieve a complete causal explanation of given sexual or aggressive behaviour, contrary to what is presumed in the discussions on how to utilise this research in practical contexts. Similarly, after analysing what proponents of utilising the behavioural sciences in practices of policymaking presume, I ask what knowledge the behavioural research provides. Is the project of behavioural public policy informed by a proper understanding of what we know on the basis of behavioural research?

Inspired by Longino, I begin by analysing what knowledge is provided by the findings in the behavioural sciences that are utilised in policy (this section). In the second step of my argument (Sect. 5) I compare the results of my analysis with knowledge claims about behavioural science made in the debates on the behavioural turn in policy in order to show that proponents of behavioural public policy (as well as many discussants of it, including philosophers) have an oversimplified understanding of the behavioural sciences. In particular, I look at prospect theory, heuristics-and-biases, and loss aversion research and I examine the knowledge these scientific approaches provide.

Prospect theory is a theory of decision-making proposed in Kahneman and Tversky (1979). It became an influential theory in cognitive psychology and a foundational one for behavioural economics. Together with research on heuristics and biases, which followed Kahneman and Tversky’s work on decision-making under risk, it is treated as the most important finding informing behavioural public policy. I decided to look closely and in detail at prospect theory, as there is a great deal of superficial understanding of what this theory is about, especially in the debates on behavioural policy, and I think that we need to come back to the theory in Kahneman and Tversky’s formulation in order to be able to understand what we can and cannot know on the basis of it. Research on loss aversion is the case study I use to show how experimental research is conducted in this field of behavioural science. I chose it as an example of experimental behavioural studies because loss aversion is treated as an important and robust finding. Furthermore, according to Kahneman (2011), “the concept of loss aversion is certainly the most significant contribution of psychology to behavioural economics” (300)*.*

### Prospect theory

Prospect theory is a theoretical proposal that accounts for experimental evidence showing that people’s “preferences systematically violate the axioms of expected utility theory” (Kahneman and Tversky 1979: p. 263). In order to make sense of the experimental findings that were not in accordance with the predictions of EUT, Kahneman and Tversky claimed that when agents face decision problems which have a structure of so-called Allais paradox^11^ they assign values to gains or losses rather than to final outcomes and these outcomes are assessed by them as gains or losses from a given reference point. Kahneman and Tversky also stated that the certainty of receiving a payoff is valued more than the expected utility of a prospect (an event x with probability p) with a higher monetary value. In addition, according to them, overweighting certainty (which they called the psychological principle), favours risk aversion in the domain of gains and risk seeking in the domain of losses. Kahneman and Tversky also noticed overweighting low tail probabilities and underweighting high tail probabilities and they argued that agents do not assign probabilities to outcomes in order to calculate the expected value of an option but instead they weight probabilities.

Prospect theory is supposed to generalise over these observations and interpretations of experimental research on decisions about monetary payoffs in risky conditions.^12^ Kahneman and Tversky envisioned the decision process itself as consisting of two phases: the phase of editing prospects and the phase of evaluating prospects.^13^ The editing phase has a character of a preliminary analysis of a prospect and it often leads to simplification of the representation of a problem. The evaluation phase is formalised in terms of two functions: a weighting function π (p) which reflects the impact which probability has on the overall value of a prospect, and a value function v(x) which assigns a subjective value to outcomes (x). The overall value of a prospect is determined in such a way that probabilities attached to each event are weighted by function π, and subjective values of each are determined by function v, which enables measurement of the distance of an event from a reference point (its subjective value). One of the characteristics of the value function is loss aversion. This is a feature of the function which determines its asymmetric S-shape and makes it steeper in the negative domain than in the positive one. Two other characteristics of the value function are: reference dependence (carriers of value—gains and losses—are defined relative to a reference point) and diminishing sensitivity (the greater the size of gains and losses the lower the marginal value of both gains and losses).

To sum up, what do we know on the basis of prospect theory? In prospect theory, decision-making is conceived as an abstract procedure (process) consisting of distinct steps (an editing phase and an evaluation phase) and operations that guide the information processing. These operations are formalised as functions assigning weight to probabilities and measuring the value of departures from a reference point. The theory provides a representation of a decision process, or in other words, the formal architecture of this process, often also called cognitive architecture. It should be noted that prospect theory does not study the *causes* of behaviour. The theory is a generalisation of the experimental studies that are interpreted under the presumption that there is a causal link between the outside information, information processing (guided by the abstract procedures), and behaviour. It is also assumed that the outside information about the probability of events (prospects) is given to decision-makers and that it triggers the information processing and the decision-making process. In Sect. 5, I show that when we account for these features of prospect theory, we can question the widely accepted claim that prospect theory offers a ‘realistic’ and ‘descriptive’ view of decision-making.

### Heuristics and biases

Proponents of behavioural policy also draw on research on heuristics. Most of them use the notion of heuristics worked out by Tversky and Kahneman (1974).^14^ The precise definition of heuristics has not been formulated in this research. It is generally claimed that heuristics are mental shortcuts or rules of thumb used under conditions of uncertainty to assess probabilities. According to Tversky and Kahneman, heuristics are used in order to formulate judgments about likelihood of events rather than as rules of decision-making. The “classical” heuristics described and analysed by Tversky and Kahneman are availability heuristics, representativeness heuristics, and anchoring heuristics.^15^ They claim that reliance on heuristics leads to systematic errors, or biases (Tversky and Kahneman 1974).^16^ The idea of heuristics has been proposed as a generalisation over experimental research which studies how people deal with tasks of assessing probabilities.^17^

What knowledge does the heuristics-and-biases approach provide? It studies how judgments about probabilities are made, in contrast to prospect theory which assumes that probabilities are given and are accessible to decision-makers. The process of decision-making itself has not been examined in this research. The research on heuristics-and-biases could be seen as the analysis of judgment formation at a preliminary stage before the decision-making takes place. The notion of heuristics is a generalisation over results of experimental testing (e.g., testing reliance on probability theory when assessing probabilities of events by agents). Heuristics are abstractions from the behaviour observed in such experiments and have a form of rules which state how information about events is processed and how it leads to a judgment about probability. These rules do not have a form of abstract procedures or processes, as they do in the case of prospect theory. Yet this research also presumes a causal link between outside information, the rules which account for it, and judgments made by agents. Below, in Sect. 5, I argue that proponents and discussants of behavioural policy seem to misunderstand that this causal link is only presumed, and not established, in behavioural research.

### Research on loss aversion

As mentioned earlier, according to prospect theory, loss aversion—a tendency to treat losses as looming more than gains—is responsible for the shape of the value function. As Tversky and Kahneman put it, loss aversion is “[t]he aggravation that one experiences in losing a sum of money appears to be greater than pleasure associated with gaining the same amount” (Tversky and Kahneman 1979: p. 273). The tendency of loss aversion, which Tversky and Kahneman observed in experiments testing expected utility theory, has been further studied experimentally and many cognitive psychologists and behavioural economists have claimed it to be robust (see: e.g. Gaechter et al. 2007; Li et al. 2012; Abdeallaoui et al. 2007; Camerer 2005; Bleichrodt et al. 2001; Booij and van de Kuilen 2006). This is one of the reasons why research on loss aversion is treated as a solid basis for policymaking.

Several types of experimental study involve loss aversion. We can group them according to the type of the context in which decisions are made by subjects.^18^ Hence, we can distinguish between studies in “thin” contexts versus “thick” contexts. In the former, the impact of monetary losses or gains on decisions is examined (see: e.g. Abdellaoui et al. 2007; Rabin and Weizsäcker 2009). In the latter cases, decisions are made in “thicker” contexts when subjects during experiments are being endowed with a certain good and decide whether to give it up, or keep it (Thaler 1980; Kahneman et al. 1990), when they make decisions about sticking to the status quo (Samuelson and Zeckhauser 1988), or when they assess progress in achieving racial equality by different racial groups (Eibach and Keegan 2006).^19^

However, the robustness of the phenomenon of loss aversion has recently been questioned. It is claimed that the phenomenon is highly dependent on the experimental design. For instance, Eldad (2018) points out that most experiments reporting loss aversion ask questions about high monetary amounts, whereas when the amounts of money that people decide about are low or moderate, the loss aversion is not observed (for a review of the literature see: Yechiam and Hochman 2013; Gal and Rucker 2018). Furthermore, experimental research that claims to identify loss aversion has mostly focused on hypothetical decisions, but when decisions are incentivised, loss aversion is observed less often, or is not observed at all (see early experimental studies such as Davidson et al. 1955; Lichtenstein 1965). Researchers also report cultural and individual differences when it comes to the occurrence of loss aversion, especially in “thicker” contexts (Apicella et al. 2014; Canessa et al. 2013; Tom et al. 2007) and point out difficulties with extrapolating results of a studied group to other groups, or to the whole population (Novemsky and Kahneman 2005).^20^

From the recent discussion on the experimental studies of loss aversion we learn that it may be a less robust phenomenon than adherents of behavioural policy believe. In Sect. 5 I indicate that this may be related to the inherent value-ladenness of how research on loss aversion is produced. I also argue that recognising how value commitments enter this research enables us to question the claim that these studies identify *cognitive* causes of behaviours.

### A brief comment on historical developments in the behavioural sciences

The behavioural research that inspired the behavioural turn in policy, and which I briefly and partly characterised above, consists of a subset of research in cognitive psychology and behavioural economics. Proponents of behavioural public policy have not clarified why the label ‘the behavioural sciences’ is used to refer to this narrow subset of behavioural research, neither whether it has affinities with the behavioural sciences programme which developed in the 1950s,^21^ or with the biological studies of behaviour, such as those that Longino (2013) discusses (cp. Plaisance et al. 2012). Exemplifying the sense in which cognitive psychology and behavioural economics are *behavioural* sciences is beyond the scope of this paper. My aim here is to argue that accounting for the origins of prospect theory, heuristics and biases, and experimental behavioural research allows us to understand better the kind of knowledge they provide. Therefore, I point out the importance of the so-called cognitive revolution and operations research for the development of studies on judgment and decision-making in cognitive psychology, prospect theory, heuristics and biases, as well as for experimental behavioural research, such as studies on loss aversion. I also discuss links between operations research and modern neoclassical economics.

In the 70s and 80s, the rapid technological advances in computer technology and cybernetics led to the rise of cognitive psychology, which included research on pattern recognition, attention, categorisation, memory, reasoning, problem solving, judgment and decision-making and language as information-processing in the mind (see Gardner 1985; Baars 1986; Laehey 1992). The computer models that inspired the developments of early cognitive psychology used complex symbols as representations that are processed in a procedural manner. Cognitive psychology was also influenced by earlier developments in operations research. Operations research entailed the study of optimisation and decision-making with the use of mathematical methods and had origins in the WWII research on optimal decision-making (Lardner 1984; Gass and Assad 2005). Operations research continued during the Cold War period and contributed to the emergence and flourishing of interdisciplinary research programmes, such as cybernetics, computer science, AI, systems engineering, as well as cognitive psychology (Mirowski 1999). It was also crucial to the further transformation of economics into a formal, abstract, mathematical science: a process that paved the way for the dominant position of economics within the social sciences (Mirowski 2002).

Historians have demonstrated compellingly that the links between economics, cognitive psychology and operations research, as well as the so-called the ‘command-control-communication-information’ research paradigm, were strong and significant (Mirowski 1999; Erickson et al. 2013). Operations research had an important influence on the behavioural sciences, including neoclassical economics and cognitive psychology; whereas cognitive psychology was consequential to the rise of the behavioural economics. Thus, all these research programmes within the behavioural sciences were highly abstract and mathematised. Furthermore, the view of what is decision-making in neoclassical economics and in cognitive psychology, as well as in behavioural economics, were not that different. Decision-making is imagined in all these research approaches as a procedure of following rules that organise information in a systematic manner. I argue that this historical work allows us to call into question the claim, widely endorsed in debates on policy applications of behavioural science, that cognitive psychology and behavioural economics provide a more descriptive and radically different account of human behaviour and decision-making than neoclassical economics (in particular EUT). I elaborate more on this point in the next section.

## The knowledge claims about behavioural research scrutinised

The above analysis is the basis for the rest of the paper, which is aimed at scrutinising the knowledge claims about the behavioural sciences prevalent in behavioural policy and in discussions accompanying it. Each subsection below discusses and questions one aspect of the view of what is known on the basis of the behavioural sciences, widely shared by proponents, critics and discussants of behavioural public policy and reconstructed in this paper (see Sect. 3 above). I draw on experimental works on loss aversion as a case study to conduct my analysis in Sects. 5.3–5.5.

### Does behavioural research reveal irrationality of behaviour?

In debates about behavioural policy it is often claimed that the behavioural sciences provide us with evidence that demonstrates human irrationality. Studies on biases in judgment and decision-making, initiated and conducted by Tversky and Kahneman, are based on the presumption that the way in which people make decisions in most contexts is flawed and irrational. This is the case because such judgement and decision-making does not ‘conform’ to the theories treated by Tversky and Kahneman as normative, such as the expected utility theory or classical logic. Does this research really reveal irrationality, though?

I start my analysis in this subsection by pointing out that scientific research cannot make this claim, as rationality and irrationality are normative categories in the light of which scientific findings are interpreted and assessed. A number of commentators have already made this point convincingly, including psychologists who have criticised Tversky and Kahneman’s research for its commitment to a notion of rationality as maximisation, consistency, statistical numeracy, and for treating it as a normative standard, or normative ideal for decision-making (Gigerenzer 1996; Lopes 1991).^22^ However, contrary to some of these critics, I do not think that alternative approaches to studying decision-making should necessarily come up with alternative concepts of rationality (such as ecological rationality proposed by Gerd Gigerenzer—see e.g.: Goldstein and Gigerenzer 2002).^23^ Instead I suggest that we need more philosophical scrutiny in uncovering the presence of value commitments, such as commitments to notions of rationality, and in understanding their epistemic role in research done in the behavioural sciences. Doing so will allow us to see that the rationality assumption not only serves as an ideal of behaviour or normative assessment of research in Tversky and Kahneman’s work, but influences it in more substantial ways.

The insights from the philosophy of science on the ways in which value commitments enter scientific research at each stage of inquiry can be helpful in understanding the role played in behavioural science by the rationality assumption (understood as maximisation, consistency, and statistical numeracy). For instance, Elisabeth Anderson offers a stylised division of the stages of research that can be influenced by values:

“(a) Researchers begin with an orientation to the background interests animating the field, (b) frame a question informed by those interests, (c) articulate a conception of the object of inquiry, (d) decide what types of data to collect, (e) establish and carry out data sampling or generation procedures, (f) analyse their data in accordance with chosen techniques, (g) decide when to stop analysing their data, and (h) draw conclusions from their analyses” (Anderson 2004: p. 11).

Anderson’s conceptualisation is useful for identifying value dimensions in the case of the behavioural sciences analysed in this text.^24^ Commitment to the norm of rationality influences the way in which research on heuristics and biases and prospect theory is conducted at almost all stages. Rationality as maximisation, whose formal treatment is given in EUT, is understood as a normative ideal for choice—it is the orientation to the background interests (stage a on Anderson’s view). This notion of rationality also has an impact on the way in which the research question is framed: what researchers study and try to explain are the discrepancies from the norm of rationality (stage b). It should be noted here that scholars of judgment and decision-making in cognitive psychology have already discussed whether this way of framing a research question may in fact have detrimental effects on research in cognitive psychology. They point out that the processes responsible for decision-making may have nothing to do with treating certain behaviour as rational or deviant (Elqayam and Evans 2011). Furthermore, the discrepancies from a norm of rationality are articulated and conceptualised as biases or deviations that are systematic (stage c). Such a conceptualisation suggests that there must be a cause of the systematic behavioural tendencies of individuals that is to be uncovered in scientific research—this conceptualisation is then important for analysing the data produced during research (stage f). The experimental data are interpreted under the presumption that decisions observed in this research are caused by information processing procedures which are different from the ones envisioned in EUT. In the analyses below I scrutinise in more detail the role of the presumption about this causal relationship.

Enthusiasts of behavioural policy who advocate reliance on behavioural research when designing policies are incorrect when they claim that this research reveals irrationality of behaviour. These claims can be accepted only as shorthand for saying that results of this research are assessed as being irrational from the point of view of a standard of rationality. This may sound obvious, but it is important to point it out because in debates surrounding behavioural public policy, emphasis is put on discussing the fact that experimental results are not in accordance with standards of rationality, such as maximisation. However, asking why rationality as maximisation is treated as a standard and why we even need such a standard to evaluate behaviour is an equally significant question to raise, especially when one intends to rely on the findings of this research in practical (policy) contexts and ‘eliminate irrationality’, which is precisely what behavioural policies often attempt to do. At the same time, the scope and character of the commitment to the notion of rationality in behavioural science is still not fully understood in the debates. As I suggest, this commitment is a form of value-ladenness of this body of behavioural science that has an important influence on how research on decision-making is framed and conducted. This value-ladenness provokes questions unrecognised in the discussion on behavioural policy. Firstly, how does one make values embedded in research explicit? Secondly, does acknowledging that value-laden research is relied on in policymaking challenge the idea of evidence-based policymaking, of which behavioural public policy is an instance?^25^

### Are some behavioural approaches more realistic and descriptive than others?

Prospect theory is treated as a more realistic and empirically-informed theory of decision-making than expected utility theory. The claim about a realistic character of prospect theory is formulated without any philosophical refinement. It seems that the ‘realistic’ status here means two things: that prospect theory is true of psychological processes (realism as a philosophical theory of scientific theories), as well as that its assumptions are more realistic or are less idealised (see e.g. Mäki 2009, 2012). My analysis in this subsection starts from the reminder that claims about such features of prospect theory are always made in contrast to EUT. If we take into consideration historical works on the origins of prospect theory and EUT (Mirowski 2002; Laehey 1992; Gass and Assad 2005) and we treat both theories as theories of information processing, it sheds new light on the contrast.

Decision-making in prospect theory is conceptualised as a slightly more complicated process than the one envisioned in expected utility theory, which prospect theory intended to replace. In EUT, the decision-making is understood only as a result of calculating probabilities of events and assigning utilities to them. In prospect theory it is an abstract procedure (process) consisting of distinct steps (editing phase and evaluation phase) and operations guiding the information processing. These operations are formalised as functions assigning weight to probabilities and measuring the value of departures from a reference point. Hence, decision-making is envisioned in prospect theory still in a highly idealised, abstract and formal way, yet as being more complex than in EUT. Seen from this perspective, prospect theory could be treated as being more ‘realistic’ than EUT, especially if it can be presumed that the mind consists of complex processes guiding decision-making.^26^ However, the extent to which prospect theory differs from EUT in respect to a realistic character in this sense is minor, especially if we recognise that both theories can be treated as theories of information processing and they differ only slightly in the way in which they represent this processing.

Prospect theory is a theoretical proposal that resulted from generalising over experimental findings. In this sense we can see why it is treated as a more empirical or ‘descriptive’ theory than EUT which had a different origin and was proposed as a theory offering a mathematical treatment of the “principle of maximisation” (von Neumann and Morgenstern 1947: p. 9). Yet most of the experimental studies that Tversky and Kahneman accounted for were studies of how choices between lotteries are made and how subjects decide about monetary payoffs in highly artificial scenarios. Treating prospect theory as a descriptive theory of how ‘people really behave’, which is the case in debates on behavioural policy, obfuscates the fact that it is a theory which generalises over such experimental studies in artificial settings and is an abstract and formal theory which provides a representation of a decision-making procedure.

I show that if we treat prospect theory and EUT as theories of information processing, then they are theories of the same type and the extent to which they differ does not justify interpreting EUT as unrealistic and prospect theory as realistic and descriptive theory of decision-making. It is important to note this, as the realistic and descriptive character of prospect theory is an argument for treating it as a model of decision-making which could be relevant in policy contexts.

### Are behavioural tendencies robust?

Loss aversion is treated as a robust phenomenon by many behavioural scientists. However, the robustness of the phenomenon of loss aversion has been recently called into question. As I mentioned in Sect. 4.3., cognitive psychologists have pointed out that loss aversion is a phenomenon that is highly dependent on experimental design and more difficult to replicate than previously thought. This is an important methodological claim which potentially may turn out to be consequential for the policy applications of this body of research, as the widely-shared beliefs about the robustness of experimental work on loss aversion justify reliance on it in policy contexts. In addition, methodological challenges such as the extrapolation problem, are already discussed by researchers themselves, as some researchers studying loss aversion have asked what reasons are there to make generalisations about reactions towards loss based on experimental studies that mostly investigate subjects’ decisions about monetary payoffs, and under what conditions can findings about a group be extrapolated to the whole population (see: e.g. Novemsky and Kahneman 2005; Canessa et al. 2013)? Below I point out other philosophical issues related to the ways in which loss and loss aversion are conceptualised and operationalised in the experimental research in question, which may further clarify the lack of robustness of experimental findings on loss aversion.

Loss is a thick concept, that is, a concept that has a descriptive but also an evaluative aspect: it is a change in the state of affairs which is assessed pejoratively. In the work of Kahneman and Tversky, losses and gains are defined as changes, negative or positive respectively, from what individuals perceive as a reference point. It is a definition that does not require the reference point to be identified, and as such, it suffices for the sake of formalisation of decision phases in prospect theory. In experimental research, however, the reference point has to be indicated and interpreted by researchers. Hence, loss is operationalised in the experiments as e.g.: giving away a good endowed to someone (e.g. Kahneman et al. 1991); getting a negative sum of money with a certain probability (e.g. Abdellaoui et al. 2007); refraining from the status quo (e.g. Samuelson and Zeckhauser 1988); or being deprived of income (e.g. Boyce, Wood and Ferguson 2016). Respectively, the reference point, in the light of which options are assessed as losses or gains is understood as: being endowed with a good, being endowed with money, being endowed with income, or status quo—which is itself further interpreted differently, for instance as having or entertaining a default option.

Hence, in order to operationalise the reference point, researchers make a value judgment about whether an event, or change of circumstances, is positive (gain) or negative (loss). Researchers come up with operationalisations of a reference point listed in the paragraph above in light of their knowledge and experience of how social, institutional and political realms work (for instance, how money functions as an institutionalised means of exchange, what the regimes of property are within which one is endowed with a good, and how a legal system defines default rules). Thus, value judgments enter the research on loss aversion: different intuitions on which change is pejorative and which is not will lead to conceptualising the term differently (for instance, for those who benefit from the status quo, refraining from it can be perceived as a loss; for those who do not benefit from the status quo, it is typically viewed as a positive change).

If research on loss aversion is less robust than claimed by proponents of behavioural policy, it means that their argument for using it for the sake of policymaking is substantially weakened. Furthermore, there are reasons to believe that the results of experimental work on loss aversion are not robust because of the inherent value-ladenness of the experimental studies. First of all, because of this value-ladenness related to the thickness of the concepts of loss and gain, researchers operationalize them differently and they may study different phenomena under the label of ‘loss aversion’. As Longino (2013) teaches us, one of the reasons why we cannot put together all five behavioural approaches to aggression and sexuality to achieve a complete causal explanation of given sexual or aggressive behaviour is the fact that the notions of aggressive behaviour and sexual behaviour are operationalised differently, and in value-laden ways. Research on loss aversion faces a similar challenge. Secondly, it may be also the case that in some experiments, researchers do not study loss aversion understood as a result of information processing in the mind (what prospect theory presumes), because the way in which they operationalise the term ‘loss’ may lead them to detect processes or phenomena as being of a social, economic, or institutional kind. Such value-ladenness may occur across behavioural research, and if so, then it poses a challenge to policy applications of experimental findings. Furthermore, if unrecognised, it may lead to confusion of cognitive processes with social or economic ones. I discuss this possibility in more detail in Sect. 5.5.

### Does behavioural experimental research provide evidence that changes in behaviour are caused by a cognitive process?

The proponents of relying on findings in the behavioural sciences in policymaking claim that these sciences enable us to identify the cognitive processes that cause changes in behaviour. It is another reason why this research is treated as policy-relevant—the claim is that behavioural policies intervene on these cognitive processes and that such interventions bring about behavioural effects. The question is whether the experimental evidence they refer to when making such claims indeed substantiates them. I have brought in experimental research on loss aversion as a case study to address this question. Does this research provide evidence that changes in behaviour are caused by a cognitive process?

One of the consequences of the underdetermination of theory by evidence is that data can be taken as evidence for a certain hypothesis only in the light of background assumptions (Longino 1990).^27^ According to Longino, background assumptions in some cases take the form of what she calls an explanatory model: “normative and somewhat general description of the sorts of items that can figure in explanations of a given sort of phenomenon and of the relationships those items can be said to bear to the phenomena being explained” (Longino 1990: p. 134)*.* Longino exemplifies her definition of an explanatory model in the following way:

in behaviourist psychology, explanations must appeal to environmental stimuli as independent variables and treat externally (extensionally) described behaviour as the variable dependent on these environmental stimuli. Explanations that describe behaviour as by means of agents’ intentions or that treat states of consciousness as independent variables do not conform to this model and are ruled out by the behaviourist program (Longino 1990: p. 135).

In behavioural experimental research, explanations appeal to a causal relation between outside information, cognitive state, and behaviour. A cognitive state, understood as a subjective valence of the state of the world, is a result of processing information about some aspects of states of the world, which causes a behavioural reaction; it is in light of such an explanatory model that data are interpreted in experiments done by behavioural scientists. For instance, in the case of studying loss aversion, subjects’ reports about their decisions regarding monetary payoffs are interpreted by behavioural researchers as being caused by processing information about the decision options (framed as gains or losses) in a way stipulated by prospect theory or alternative theories in cognitive psychology (Sect. 6).

Yet this causal relationship between a cognitive state and behaviour comes from the background assumption. This assumption is needed to make sense of experimental data. Hence, the causal link between the cognitive state and behaviour is presumed to hold. It is not ‘discovered’ in behavioural research, as is often believed in the debates on behavioural policy. This means that this body of research does not provide knowledge of cognitive causes on which one could intervene, contrary to what proponents of behavioural policies claim and to how they imagine ‘mechanisms’ through which policies impact behaviour: by causing a change in behaviour after altering a cognitive state of an agent.

### Does behavioural experimental research identify cognitive processes?

As we have seen, prospect theory conceptualises the process of decision-making as consisting of two phases: (1) editing information about outcomes as loss or gain and (2) assigning values to them. During the second phase, subjects assign values in such a way that losses loom larger than gains and therefore subjects’ behaviour displays loss aversion. However, researchers debate whether it is the right way to conceptualise the cognitive decision processes that triggers behaviours.

For example, some propose an attention-based account of decision-making as an alternative and claim that “under loss aversion the (…) [process assumed to be affected by losses-MM] involves the translation of objective outcomes into subjective valences. Under the attention-based account, losses reduce random noise and increase the sensitivity of choices to the incentive structure of the task” (Yechiam and Hochman 2013: p. 214).

Kahneman himself in his more recent work has begun to research further how the decision process leading to loss aversion could be conceptualised differently, in order to account for the evidence challenging the robustness of loss aversion. In his work with Novemsky, he focused on decisions made in so-called riskless contexts—mainly the ones in which subjects decide about goods endowed to them, such as primary exchange goods like money (Novemsky and Kahneman 2005). Novemsky and Kahneman claim that during experiments about deciding how to allocate money, subjects make decisions influenced by their initial intentions about how to spend money—so-called budgeting intentions. Novemsky and Kahneman further argue that under such conditions, if goods are exchanged as intended, they are not evaluated as losses. This leads them to conclude that “the coding of outcomes as gains and losses depends on the agent’s intentions and not only on the objective state of affairs at the moment of decision” (Novemsky and Kahneman 2005: p. 127).

We see that while researchers try to modify or come up with an alternative cognitive ‘architecture’ of decision-making processes, they very often import their intuitions about social and economic realms in order to formulate hypotheses about these cognitive processes. For instance, they refer to the notions of budget, or incentive structure, when they formulate hypotheses about how the cognitive architecture works. Their intuitions about economic and social realities clearly inform the ways in which they imagine cognitive processes and when they try to identify a cognitive process which leads to loss aversion. The notion of budgeting intentions illustrates this.

For example, the experience of social and economic practice of budgeting leads Novemsky and Kahneman to suggest that there are cognitive procedures which resemble this practice, or are analogous to it. Furthermore, Novemsky and Kahneman also generalise over the specific research on decisions about money and budgeting intentions, which is influenced by their intuitions about social and economic reality. When they come up with generalisations, they decontextualise the findings of this particular research on decisions about allocation of money and present them as demonstrating a general process of the cognitive coding of information. As a result of this decontextualisation, the presumptions in light of which they formulate their hypothesis about cognitive processes (e.g. what they presume about money and budgeting practices) and the specificity of the case completely disappear from sight and are difficult to detect by those who familiarise themselves only with the final result of this research which reports detecting a way of coding of information about losses and gain.

The analysis of the research on loss aversion allows us to realise that at least in some cases, the way in which researchers come up with theoretical proposals on how information is being processed is influenced by and abstracted from the details of the social or economic contexts and background conditions in which the analysed decisions take place. In this way, again, value (social and economic) considerations enter this research and it may be the case that we are dealing here with a projection of social and economic processes on the cognitive ones. Hence, processes which are identified in this research may not be cognitive, or at least not purely cognitive. For this reason, the claim made by the proponents of behavioural policy that behavioural research identifies ‘psychological processes underlying human behaviour’ can be questioned. As the consequences of such a value-ladenness of behavioural science, such as treating economic or social processes as cognitive ones, are neglected in the debates about behavioural policy, this may lead to misunderstanding why and when these policies are, or are not effective.

## Conclusion

Above I analysed the knowledge claims about the behavioural sciences prevalent among policymakers as well as in the academic debates on behavioural public policy. Then I looked at the knowledge provided by the behavioural sciences that are referred to in order to design and justify behavioural policy, and I compared it with the knowledge claims made by proponents of the behavioural turn in policy. I argued that the subset of the behavioural sciences I analysed, and which is used in behavioural policy (prospect theory, heuristics-and-biases research, studies on loss aversion) does not, and cannot, reveal irrationality of behaviour. However, at many stages of scientific inquiry, this research is strongly influenced and driven by the commitment to a certain notion of rationality. This research is also value-laden in several other ways discussed throughout the text. Uncovering value commitments of the behavioural sciences allows us to understand better why behavioural experimental research is less robust than is claimed, as the case of studies of loss aversion shows. It also enables us to see that sometimes this research may identify changes in behaviour that are not caused by cognitive factors, but rather by social or economic ones. Furthermore, the causes of individual behaviour by cognitive processes are not actually revealed in the research, but they are presumed in the background assumptions (in the explanatory model, how Longino calls it). Finally, prospect theory envisions decision-making as a slightly more complex process than the expected utility theory. However, it is questionable whether it is a more ‘realistic’ theory, as it still remains highly abstract and formal and shares with EUT the vision of decision-making as information processing.

As my analysis suggests, the claims about the behavioural sciences widely shared in the debates on behavioural policy are not substantiated in behavioural research. They seem to be a view of scientific knowledge about behaviour that proponents of behavioural policy would wish to have in order to change individual behaviours in a way they imagine. I do not want to suggest that their view on behavioural science results from a manipulation of scientific research. Rather, it contains elements of background assumptions (e.g. on causal relationships), which are mistakenly treated as findings, or it does not recognise the proper role which standards of rationality play in behavioural research. An analysis in philosophy of science that I advanced here allowed this to be detected. Yet it also raises further questions. Why do the knowledge claims about the behavioural sciences, which I have shown to be problematic, prevail in policymaking? What role, apart from providing alleged solutions, does behavioural science play in defining and diagnosing challenges to be addressed and tackled in policy? As behavioural science is value-laden, are the values embedded in this research an important factor for why and how this research is so often applied to policy contexts? Does the discussion on moral and political aspects of behavioural policy and nudging, mentioned in the introduction, need to be rethought because it presumes the knowledge claims I scrutinised? Finally, what is the relationship between the behavioural research analysed here and the widely accepted idea among proponents of behavioural policy that policymaking should aim at a modification of the behaviours of individuals? I hope that this article will convince scholars about the importance of asking such questions. The proper understanding of what is known on the basis of behavioural science is an indispensable starting point in order to analyse the relationship of behavioural research with policy (power) practices which utilise it.

## Footnotes

1 For instance, scholars have discussed whether behavioural policy is a form of manipulation of citizens’ choices (Bovens 2009), whether it infringes on their autonomy (Cohen 2013), or whether it is a form of paternalism (Veetil 2011; Hausman and Welch 2010). Political and social theorists have debated whether behavioural policy is a manifestation of transformations within a neoliberal state (McMahon 2015; Jones et al. 2013), or whether it is compatible with existing democratic legal institutions (Lepenies and Małecka 2015; Allemano and Sibony 2015).

2 Evidence-based policy is an approach to policymaking that advocates designing policy solutions on the basis of the best available scientific evidence. Its proponents suggest that scientific research should be consulted in order to diagnose a problem which policymakers address and to test policy proposals in experimental settings during randomised controlled trials. The idea of experimental tests of policies is closely related to the view on policymaking as finding efficient and effective interventions (so called ‘what works’ policymaking), which was promoted by the new public management approach (Head 2013). Adherents of such an approach believe that it allows us to limit or eliminate “ideologically-based decision making” (Banks 2009, see also: Cartwright and Hardie 2012). An evidence-based approach to policymaking has been heavily criticized for the positivist, empiricist worldview that underpins the theory and practice of the approach and fails to address key elements of the policymaking process (Greenhalgh and Russell 2009), for promoting a colonial discourse and material relations of power that continue from the American‐European colonial era, for instance in the field of education (Shahjahan 2011), and for its links with new managerialism that undermine progressive policies (Davies 2003).

3 Nudging, in the eyes of its advocates, is a public policy tool that allows for steering people’s behaviour towards welfare-promoting outcomes while not limiting their freedom of choice, usually by changing the context of their choice (i.e., by changing ‘choice architecture’, as it is often called in the ‘nudge’ literature). Proponents of nudging try to defend it, normatively and politically, using the position of a doctrine called libertarian paternalism which states that shaping people’s choices by governments is justifiable once people can easily refrain from such an influence.

4 So-called nudge, or behavioural, units are being established worldwide: e.g. in the UK, the US, Germany, France, the Netherlands, Australia, Japan and Singapore, as well as at the World Bank and among different teams within the United Nations, at the OECD, and the European Commission (Joint Research Centre).

5 Status quo bias is a tendency to place higher values on options perceived as the status quo. Tversky and Kahneman 1991 argue that phenomena such as the endowment effect and status quo bias, observed in experimental settings, may be explained as consequences of loss aversion.

6 This policy illustrates how nudging is supposed to impact people’s choices—it changes the context of their choice by setting the default, but it still gives them the opportunity to opt out. In this sense, it has been claimed that nudges are paternalistic and libertarian at the same time. The choice architecture—in this case, the default rule—influences people’s choices in directions set up by policymakers, but people can always opt out from the default option and hence ‘choose freely’.

7 The availability heuristic is a mental shortcut used in situations in which people assess the frequency of a class or probability of an event based on how easily instances or occurrence can be brought to mind. The affect heuristic is a mental shortcut used when making automatic decisions, whereby one relies heavily upon one's emotional state during decision-making.

8 However, there is evidence that shocking health warnings do not have discouraging effect on all people, in particular not on those who are highly dependent on nicotine (Shadel et al. 2019). In fact, the effects of many behavioural public policies, when tested in natural or laboratory experiments, show discrepancy with the predictions formulated by the proponents of these policies; usually effects of behavioural policies vary among different groups of people and across different contexts of policy’ implementation. The usual remedy for this challenge suggested by proponents of behavioural policy is to do more behavioural research and to conduct more tests of behavioural policies. However, such a recommendation contradicts the pragmatic justification given for bringing the behavioural sciences into policymaking: to design effective policies in a way which allows for the costs of policymaking to be limited. It should be also pointed out that these challenges do not lead to revising knowledge claims about research in the behavioural sciences that I analyse (Sect. 3) and criticise (Sect. 5).

9 Expected utility theory was introduced to modern economic theorising by John von Neumann and Oskar Morgenstern in their seminal book *Theory of Games and Economic Behaviour* (von Neumann and Morgenstern 1944). The theory states that when making decisions under conditions of risk, a decision-maker compares the expected utility of options (utility value of options multiplied by the probability of getting them) and opts for the one that has the highest expected utility. Von Neumann and Morgenstern provided the mathematical, axiomatic treatment of the thus understood maximisation of the expected utility. Alternative axiomatisations of EUT were proposed by Marschak (1950) and Herstein and Milnor (1953). Savage (1954) developed the axiomatisation of the subjective expected utility theory under conditions of uncertainty.

10 However, there is variation in the way in which proponents of behavioural policy decide to account for biases. They sometimes advocate the elimination of biases through policy, but for the most part they propose ‘exploiting’ the biases in order to achieve the desired policy outcomes (e.g. in public health nudge policies, such as the shocking health warnings mentioned above). The latter way of using scientific findings about behavioural tendencies deemed to be biases is difficult to reconcile with the liberal justification for behavioural policy provided by its advocates. It demonstrates, however, that behavioural policy is first and foremost a behaviour technique. Scientific findings are supposed to inform policymakers how to design techniques of behavioural change that will lead to the desired changes in behaviours.

11 The Allais paradox is a name for experimental results that demonstrated the inconsistency of the actual choices made during experiments with the predictions of EUT in experimental scenarios in which participants chose between monetary payoffs received with probability of 1 (certainty), or probabilities slightly less than 1. Cf. Allais (1953).

12 Risk is understood to be an event, the probability of which is known, which differentiates it from uncertainty—an event which occurs with unknown probability.

13 Major operations of the editing phase are: coding, combination, segregation, cancellation, simplification, or detection of dominance (Kahneman and Tversky 1979: pp. 274–275). For example, segregation leads to representation of a problem in such a way that the risky component of a prospect is separated from the riskless one; cancellation consists in discarding shared components and focusing only on those, which distinguish prospects.

14 Some authors also make reference to the rival notion proposed by Gerd Gigerenzer. For a review and discussion see: Grune-Yanoff and Hertwig (2016). In this subsection I focus on Tversky and Kahneman’s proposal as it is the most influential within policy and it directly inspired the nudging agenda. Gigerenzer's discussion with Tversky and Kahneman concerns the claims that reliance on heuristics leads to mistakes (biases). Gigerenzer argues that heuristics have an important role in what he calls “adaptive thinking” (see: Gigerenzer 2000). They enable fast and frugal decision-making (see: Gigerenzer, Todd & ABC Research Group 1999), whereas decisions based on heuristics do not result in mistakes, but are rational in the specific sense. (Gigerenzer introduces another understanding of rationality that he calls “ecological rationality”—see: Goldstein and Gigerenzer 2002).

15 A representativeness heuristic is a rule of thumb which people use to evaluate the probability of an event’s belonging to a class by the degree to which an event resembles a typical member of that class. An anchoring heuristic is a mental shortcut used to assess probabilities by reference to initial value and by adjustments to that value in order to reach the estimate. Gradually, Tversky and Kahneman and their collaborators have identified and described more heuristics, such as: simulation heuristics (see: Kahneman and Tversky 1982) and affect heuristics (Slovic et al. 2007).

16 For instance, biases that concern insensitivity to prior probability of outcomes, insensitivity to sample size, misconceptions of chance, insensitivity to predictability, illusion of validity, biases in evaluation of conjunctive and disjunctive event (Tversky and Kahneman 1974).

17 An example of this is the Linda problem, in which subjects are provided with a summary description of a ‘liberal-sounding’ individual before being asked to judge whether it is more probable that she is a bank teller or a feminist bank teller (Tversky and Kahneman 1982). In many other experiments people similarly deal with tasks of probability assessments which include scenarios often embedded in participants’ typical, or everyday experience, such as estimating the number of men or women on the list of names which participants heard, when lists include more female, or male names of famous personalities, respectively (Tversky and Kahneman 1973); assigning probabilities to ten possible outcomes of the diplomatic journey of president Nixon to China and to the Soviet Union in 1972 (Fischhof and Beyth 1975).

18 By ”thicker” I understand these cases in which loss aversion is studied in contextually richer contexts than the ones in which one decides about monetary payoffs only (e.g. when business executives are asked about decisions, involving monetary losses, or gains, that they make in the context of their practice of running an enterprise, such as starting a legal dispute—e.g. Swalm 1966; or when the very concept of loss is being interpreted not as expressible in monetary terms, but as loss of a position in a social structure—e.g. Eibach and Keegan 2006).

19 In the “thin” experiments that involve decisions about monetary gambles, what is usually studied is whether people exhibit loss aversion: the phenomenon itself is examined—whether it occurs and persists. The decisions made in “thicker” contexts—like being endowed with a good and giving it up, or sticking to the status quo—are either studied when the hypothesis of aversion towards losses is tested, or these decisions are explained by loss aversion.

20 This observation echoes a general discussion initiated by the seminal article by Henrich et al. (2010) that points out challenges with extrapolating findings in the behavioural sciences based on samples drawn from so-called Western, educated, industrialised, rich, and democratic (WEIRD) societies.

21 The idea of the behavioural sciences, and the term itself, had been proposed in the 1950s in the United States. The Ford Foundation was especially important for development of the behavioural sciences. The Foundation was interested in supporting socially and policy relevant social sciences that would provide knowledge about the challenges faced by post-War American society. It initiated a programme designed to study “individual behaviour and human relations” that came to be known as the behavioural sciences programme. The programme was an interdisciplinary research endeavour, aimed at going beyond the existing boundaries of disciplines such as sociology, anthropology, psychology, biology, economics, geography, law, psychiatry, and political science, and to establish a unifying theory of human behaviour that would explain the main mechanisms of people’s behaviour (Miller 1955; Berelson and Steiner 1964). The term ‘behavioural sciences’ was proposed for this research project, in order to stress the ‘scientific’ orientation of the social sciences supported by the Foundation (and differentiate them from the “polemical, speculative and pre-scientific strands of social science” (Pooley and Solovey 2010: p. 211)), and to avoid associations with socialism.

22 See also: Małecka (2020) on how the notion of rationality is understood in this research.

23 See also: Małecka (2020) who elaborates on the possibility of getting rid of a commitment to a notion of rationality in research on individual decision-making.

24 I rely on Anderson’s conceptualisation here, but most authors who systematically analyse the presence of values in science distinguish between different stages of inquiry at which values enter scientific research (cf. Longino 1990; Douglas 2016; Elliott 2017).

25 Nancy Tuana’s work on embedding philosophers and philosophers of science in practices of interdisciplinary and policy relevant science offers some guidance on approaching these questions (Tuana 2010, 2013). See also suggestions on how to make values embedded in the behavioural sciences explicit in order to enable democratic control of behavioural public policy (Lepenies and Małecka 2019).

26 Yet it is not clear whether prospect theory, by slightly complicating representation of procedures of information processing, identifies cognitive processes responsible for decision-making. At least it is a question that can be investigated independently from the issue of ‘realism’ of prospect theory, as the scientific realism of a theory does not concern the extent to which a theory is complicated, but whether the theoretical terms refer to entities existing in the world, or whether theories are true (see e.g.: Psillos 1999).

27 It also means that, in principle, the same data can be treated as evidence for different hypotheses, including conflicting hypotheses (Longino 1990: p. 42). Experimental research in cognitive science is a good illustration of this, as for example research on loss aversion analysed in this and next subsection.

## References

[CR1] Abdellaoui M, Bleichrodt H, Paraschiv C (2007). Loss aversion under prospect theory: A parameter-free measurement. Management Science.

[CR2] Alemanno A, Sibony AL (2015). Nudge and the law: A European perspective.

[CR3] Allais M (1953). Le comportement de l’hommerationneldevant le risque: Critique des postulatsetaxiomes de l’ecoleamericaine. Econometrica.

[CR4] Anderson E (2004). Uses of value judgments in science: A general argument, with lessons from a case study of feminist research on divorce. Hypatia.

[CR5] Angner E (2012). A course in behavioral economics.

[CR6] Apicella CL, Azevedo EM, Christakis NA, Fowler JH (2014). Evolutionary origins of the endowment effect: Evidence from hunter-gatherers. American Economic Review.

[CR7] Banks G (2009). Evidence-based policy making: What is it? How do we get it?.

[CR8] Baars BJ (1986). The cognitive revolution in psychology.

[CR9] Berelson, B., & Steiner, G. A. (1964). Human behavior: An inventory of scientific findings.

[CR10] Biddle JB (2018). “Antiscience Zealotry”? Values, epistemic risk, and the GMO debate. Philosophy of Science.

[CR11] Bleichrodt H, Pinto JL, Wakker PP (2001). Making descriptive use of prospect theory to improve the prescriptive use of expected utility. Management Science.

[CR12] Booij AS, Van de Kuilen G (2009). A parameter-free analysis of the utility of money for the general population under prospect theory. Journal of Economic Psychology.

[CR13] Bovens, L. (2009). The ethics of nudge. In *Preference change* (pp. 207–219). Springer, Dordrecht.

[CR14] Boyce, Ch. J., Wood, A. M., & Ferguson E. (xxxx) Individual differences in loss aversion: Conscientiousness predicts how life satisfaction responds to losses versus gains in income. *Personality and Social Psychology Bulletin 42*(4):471–484. 10.1177/0146167216634060 26960673

[CR15] Camerer, C. F. & Loewenstein, G. (2004). Behavioural economics: past, present, future. In Camerer C. F., Loewenstein G. & Rabin M. (Eds.) *Advances in behavioural economics* (pp. 3–51). Russell Sage Foundation.

[CR16] Camerer C (2005). Three cheers—psychological, theoretical, empirical—for loss aversion. Journal of Marketing Research.

[CR17] Canessa N, Crespi C, Motterlini M, Baud-Bovy G, Chierchia G, Pantaleo G, Tettamanti M, Cappa SF (2013). The functional and structural neural basis of individual differences in loss aversion. Journal of Neuroscience.

[CR18] Cartwright N, Hardie J (2012). Evidence-based policy: A practical guide to doing it better.

[CR19] Chetty R (2015). Behavioral economics and public policy: A pragmatic perspective. American Economic Review.

[CR21] Cohen S (2013). Nudging and informed consent. The American Journal of Bioethics.

[CR22] Davidson, D., Suppes, P., & Siegel, S. (1955). Some experiments and related theory on the measurement of utility and subjective probability. Library of Congress.

[CR23] Davies B (2003). Death to critique and dissent? The policies and practices of new managerialism and of'evidence-based practice'. Gender and Education.

[CR24] Douglas H (2000). Inductive risk and values in science. Philosophy of Science.

[CR25] Douglas H (2009). Science, policy, and the value-free ideal.

[CR26] Douglas H, Humpreys P (2016). Values in science. The Oxford handbook of philosophy of science.

[CR27] Dupré J (2007). Fact and value.

[CR28] Eibach RP, Keegan T (2006). Free at last? Social dominance, loss aversion, and white and black Americans' differing assessments of racial progress. Journal of Personality and Social Psychology.

[CR29] Elliott KC (2017). A tapestry of values: An introduction to values in science.

[CR30] Elqayam S, Evans JSB (2011). Subtracting “ought” from “is”: Descriptivism versus normativism in the study of human thinking. Behavioral and Brain Sciences.

[CR31] Erickson P, Klein JL, Daston L, Lemov R, Sturm T, Gordin MD (2013). How reason almost lost its mind: The strange career of Cold War rationality.

[CR32] Finucane ML, Alhakami A, Slovic P, Johnson SM (2000). The affect heuristic in judgments of risks and benefits. Journal of Behavioral Decision Making.

[CR33] Fischhof B, Beyth R (1975). “I knew it would happen”: Remembered probabilities of once- future things. Organizational Behavior and Human Performance.

[CR34] Gächter, S., Johnson, E. J., & Herrmann, A. (2007). Individual-level loss aversion in riskless and risky choices.

[CR35] Gal D, Rucker DD (2018). The loss of loss aversion: Will it loom larger than its gain?. Journal of Consumer Psychology.

[CR36] Gardner, H. (1985). The mind's new science: A history of the cognitive revolution. Basic books.

[CR118] Gass, S. I., & Assad, A. A. (2005). *An annotated timeline of operations research: An informal history* (Vol. 75). Springer.

[CR37] Gigerenzer G (1996). On narrow norms and vague heuristics: A reply to Kahneman and Tversky. Psychological Review.

[CR38] Gigerenzer G (2000). Adaptive thinking: Rationality in the real world.

[CR39] Gigerenzer G, Todd P, ABC Research Group (1999). Simple heuristics that make us smart.

[CR40] Goldstein D, Gigerenzer G (2002). Models of ecological rationality: The recognition heuristic. Psychological Review.

[CR41] Greenhalgh T, Russell J (2009). Evidence-based policymaking: A critique. Perspectives in Biology and Medicine.

[CR42] Grüne-Yanoff T (2016). Why behavioural policy needs mechanistic evidence. Economics and Philosophy.

[CR43] Grüne-Yanoff T, Hertwig R (2016). Nudge versus boost: How coherent are policy and theory?. Minds and Machines.

[CR44] Guala F, Mittone L (2015). A political justification of nudging. Review of Philosophy and Psychology.

[CR45] Hansen PG, Jespersen AM (2013). Nudge and the manipulation of choice: A framework for the responsible use of the nudge approach to behaviour change in public policy. European Journal of Risk Regulation.

[CR46] Hansen PG (2016). The definition of nudge and libertarian paternalism: Does the hand fit the glove?. European Journal of Risk Regulation.

[CR47] Hausman DM, Welch B (2010). Debate: To nudge or not to nudge. Journal of Political Philosophy.

[CR48] Head BW (2013). Evidence-based policymaking-speaking truth to power?. Australian Journal of Public Administration.

[CR49] Henrich J, Heine SJ, Norenzayan A (2010). The weirdest people in the world?. Behavioral and Brain Sciences.

[CR50] Herstein IN, Milnor J (1953). An axiomatic approach to measurable utility. Econometrica, Journal of the Econometric Society.

[CR51] Intemann K (2001). Science and values: Are value judgments always irrelevant to the justification of scientific claims?. Philosophy of Science.

[CR52] Jolls Ch, Sunstein C, Thaler R (1998). A behavioral approach to law and economics. Stanford Law Review.

[CR53] Jones R, Pykett J, Whitehead M (2013). Changing behaviours: On the rise of the psychological state.

[CR54] Kahneman D, Tversky A (1979). Prospect theory: An analysis of decision under risk. Econometrica.

[CR55] Kahneman D, Tversky A, Kahneman D, Slovic P, Tversky A (1982). Simulation heuristic. Judgment under uncertainty: Heuristics and biases.

[CR56] Kahneman D, Knetsch JL, Thaler RH (1991). The endowment effect, loss aversion, and status quo bias. Journal of Economic Perspectives.

[CR57] Kahneman D (2011). Thinking, fast and slow.

[CR58] Keller E, Longino H (1996). Feminism and science.

[CR60] Larnder H (1984). OR forum—the origin of operational research. Operations Research.

[CR61] Leahey TH (1987). A history of psychology: Main currents in psychological thought.

[CR62] Lepenies R, Małecka M (2015). The institutional consequences of nudging–nudges, politics, and the law. Review of Philosophy and Psychology.

[CR63] Lepenies R, Małecka M, Beck S, Strassheim H (2019). Behaviour change: Extralegal, apolitical, scientistic?. Handbook of behaviour change and public policy.

[CR64] Li YJ, Kenrick DT, Griskevicius V, Neuberg SL (2012). Economic decision biases and fundamental motivations: How mating and self-protection alter loss aversion. Journal of Personality and Social Psychology.

[CR65] Lichtenstein S (1965). Bases for preferences among three-outcome bets. Journal of Experimental Psychology.

[CR66] Longino HE (1990). Science as social knowledge: Values and objectivity in scientific inquiry.

[CR67] Longino HE (2013). Studying human behavior: How scientists investigate aggression and sexuality.

[CR68] Lopes LL (1991). The rhetoric of irrationality. Theory and Psychology.

[CR69] McMahon J (2015). Behavioral economics as neoliberalism: Producing and governing homo economicus. Contemporary Political Theory.

[CR70] Mäki, U. (2009). Realistic realism about unrealistic models. The Oxford handbook of philosophy of economics.

[CR72] Mäki U (2012). Realism and antirealism about economics. Philosophy of Economics.

[CR73] Małecka M (2020). The normative decision theory in economics: A philosophy of science perspective. The case of the expected utility theory. Journal of Economic Methodology.

[CR74] Marchionni C, Reijula S (2019). What is mechanistic evidence, and why do we need it for evidence-based policy?. Studies in History and Philosophy of Science Part A.

[CR75] Marschak J (1950). Rational behavior, uncertain prospects, and measurable utility. Econometrica Journal of the Econometric Society.

[CR76] Miller JG (1955). Toward a general theory for the behavioral sciences. American Psychologist.

[CR77] Mirowski P (1999). Cyborg agonistes: Economics meets operations research in mid-century. Social Studies of Science.

[CR78] Mirowski P (2002). Machine dreams: Economics becomes a cyborg science.

[CR79] Novemsky N, Kahneman D (2005). The boundaries of loss aversion. Journal of Marketing Research.

[CR80] Oliver A (2013). Behavioural public policy.

[CR81] Peters E, Fischhoff B, Brewer L, Downs J (2011). Affect and emotion. Communicating risks and benefits: An evidence-based user’s guide.

[CR82] Plaisance K, Reydon T (2012). Philosophy of behavioural psychology.

[CR83] Pooley J, Solovey M (2010). Marginal to the revolution: The curious relationship between economics and the behavioral sciences movement in mid-twentieth-century America. History of Political Economy.

[CR84] Psillos, S. (1999), Scientific realism. London Routledge

[CR85] Rabin M, Weizsäcker G (2009). Narrow bracketing and dominated choices. American Economic Review.

[CR86] Rudner R (1953). The scientist qua scientist makes value judgments. Philosophy of Science.

[CR87] Samuelson W, Zeckhauser R (1988). Status quo bias in decision making. Journal of Risk and Uncertainty.

[CR88] Savage LJ (1954). The foundations of statistics.

[CR89] Shadel WG, Martino SC, Setodji CM, Dunbar M, Scharf D, Creswell KG (2019). Do graphic health warning labels on cigarette packages deter purchases at point-of-sale? An experiment with adult smokers. Health Education Research.

[CR91] Shahjahan RA (2011). Decolonizing the evidence-based education and policy movement: Revealing the colonial vestiges in educational policy, research, and neoliberal reform. Journal of Education Policy.

[CR116] Shafir E (2012). The Behavioral Foundations of Public Policy.

[CR92] Slovic P, Peters E, Finucane ML, MacGregor DG (2005). Affect, risk, and decision making. Health psychology.

[CR93] Slovic P, Finucane M, Peters E, MacGregor D (2007). The affect heuristic. European Journal of Operational Research.

[CR94] Sunstein C, Thaler R (2003). Libertarian paternalism is not an oxymoron. The University of Chicago Law Review.

[CR95] Sunstein CR (2016). The ethics of influence: Government in the age of behavioral science.

[CR96] Swalm RO (1966). Utility theory-insights into risk taking. Harvard Business Review.

[CR97] Thaler R (1980). Toward a positive theory of consumer choice. Journal of Economic Behavior and Organization.

[CR98] Thaler RH (2000). From homo economicus to homo sapiens. The Journal of Economic Perspectives.

[CR99] Thaler RH, Sunstein CR (2009). Nudge: Improving decisions about health, wealth, and happiness.

[CR100] Tom SM, Fox CR, Trepel C, Poldrack RA (2007). The neural basis of loss aversion in decision-making under risk. Science.

[CR101] Tuana N (2010). Leading with ethics, aiming for policy: New opportunities for philosophy of science. Synthese.

[CR102] Tuana N (2013). Embedding philosophers in the practices of science: Bringing humanities to the sciences. Synthese.

[CR103] Tversky A, Kahneman D (1973). Availability: A heuristic for judging frequency and probability. Cognitive Psychology.

[CR104] Tversky A, Kahneman D (1974). Judgment under uncertainty: Heuristics and biases. Science, New Series.

[CR105] Tversky A, Kahneman D, Kahneman D, Slovic P, Tversky A (1982). Judgements of and by representativeness. Judgment under uncertainty: heuristics and biases.

[CR106] Tversky A, Kahneman D (1991). Loss aversion in riskless choice: A reference-dependent model. The Quaterly Journal of Economics.

[CR107] Van Bavel JJ, Baicker K, Boggio PS, Capraro V, Cichocka A, Cikara M, Drury J (2020). Using social and behavioural science to support COVID-19 pandemic response. Nature Human Behaviour.

[CR108] Von Neumann J, Morgenstern O (1944). Theory of games and economic behavior.

[CR109] Veetil VP (2011). Libertarian paternalism is an oxymoron: An essay in defence of liberty. European Journal of Law and Economics.

[CR111] Weimer DL, Vining AR (2017). Policy analysis: Concepts and practice.

[CR112] Wilkinson N, Klaes M (2012). An introduction to behavioral economics.

[CR113] Wylie A, Nelson LH, Kincaid H, Dupre J, Wylie A (2007). Coming to terms with the value (s) of science: Insights from feminist science scholarship. Value-free science? Ideals and illusions.

[CR114] Yechiam E (2018). Acceptable losses: The debatable origins of loss aversion. Psychological Research Psychologische Forschung.

[CR115] Yechiam E, Hochman G (2013). Loss-aversion or loss-attention: The impact of losses on cognitive performance. Cognitive Psychology.

